# Adherence to Extended Venous Thromboembolism Prophylaxis and Outcomes After Complex Gastrointestinal Oncologic Surgery

**DOI:** 10.1245/s10434-023-13677-z

**Published:** 2023-06-20

**Authors:** Michail N. Mavros, Lauren A. Johnson, Mario Schootman, Sonia T. Orcutt, Cheng Peng, Bradley C. Martin

**Affiliations:** 1 Department of Surgery, College of Medicine, University of Arkansas for Medical Sciences, Little Rock, AR USA; 2 Department of Internal Medicine, College of Medicine, University of Arkansas for Medical Sciences, Little Rock, AR USA; 3 Division of Pharmaceutical Evaluation and Policy, University of Arkansas for Medical Sciences, Little Rock, AR USA

## Abstract

**Background:**

Clinical guidelines recommend extended venous thromboembolism (VTE) prophylaxis for cancer patients after major gastrointestinal (GI) operations. However, adherence to the guidelines has been low, and the clinical outcomes not well defined.

**Methods:**

This study retrospectively analyzed a random 10 % sample of the 2009–2022 IQVIA LifeLink PharMetrics Plus database, an administrative claims database representative of the commercially insured population of the United States. The study selected cancer patients undergoing major pancreas, liver, gastric, or esophageal surgery. The primary outcomes were 90-day post-discharge VTE and bleeding.

**Results:**

The study identified 2296 unique eligible operations. During the index hospitalization, 52 patients (2.2 %) experienced VTE, 74 patients (3.2 %) had postoperative bleeding, and 140 patients (6.1 %) had a hospital stay of at least 28 days. The remaining 2069 operations comprised 833 pancreatectomies, 664 hepatectomies, 295 gastrectomies, and 277 esophagectomies. The median age of the patients was 49 years, and 44 % were female. Extended VTE prophylaxis prescriptions were filled for 176 patients (10.4 % for pancreas, 8.1 % for liver, 5.8 % for gastric cancer, and 6.5 % for esophageal cancer), and the most used agent was enoxaparin (96 % of the patients). After discharge, VTE occurred for 5.2 % and bleeding for 5.2 % of the patients. The findings showed no association of extended VTE prophylaxis with post-discharge VTE (odds ratio [OR], 1.54; 95 % confidence interval [CI], 0.81–2.96) or bleeding (OR, 0.72, 95 % CI, 0.32–1.61).

**Conclusions:**

The majority of the cancer patients undergoing complex GI surgery did not receive extended VTE prophylaxis according to the current guidelines, and their VTE rate was not higher than for the patients who received it.

Venous thromboembolism (VTE) is a major cause of morbidity and mortality worldwide, imposing major financial impacts on health care systems.^1,2^ Several scoring systems have been developed to risk-stratify patients for VTE, and the most widely scoring adopted is the modified Caprini Risk Assessment Model. This model assigns points based on patient age, comorbidities, and surgery type, and has been externally validated with general surgery patients.^3^ Cancer patients requiring major open surgery almost invariably have a modified Caprini score of 5 or higher, indicating high VTE risk. In fact, studies using venography for VTE screening have reported an incidence reaching 37 % among postoperative patients with cancer.^4^

In 2012, the American College of Chest Physicians (ACCP) released the ninth edition of their Evidence-Based Clinical Practice Guidelines, which recommended extended-duration postoperative pharmacologic prophylaxis (4 weeks) with low-molecular-weight heparin (LMWH) over limited-duration prophylaxis for patients undergoing abdominal or pelvic surgery for cancer (recommendation grade 1B).^5^ These guidelines have been widely endorsed, including endorsement by the American Society of Hematology and the International Society of Thrombosis and Hematosis, and similar guidelines have been published since by the American Society of Clinical Oncology.^6–9^ These recommendations have been largely based on evidence from three randomized controlled trials (RCTs), which demonstrated that among patients undergoing major abdominal or pelvic surgery for cancer, extended VTE prophylaxis with LMWH decreased the incidence of postoperative VTE (compared with in-hospital prophylaxis) without increasing the risk of bleeding or mortality.^10–13^

In real life, however, LMWHs are costly and administered by daily self-injections, limiting patient adherence and satisfaction.^13,14^ This in turn limits surgeons’ compliance with the guidelines to a low of 28 % to 45 %, with surgeons expressing concerns about indications (perceived low risk of VTE and higher risk of bleeding), cost, and patient adherence.^15–18^ In this context, we used a nationwide administrative claims database to document adherence to extended VTE prophylaxis after complex GI cancer surgery and to assess whether filling an extended VTE prophylaxis prescription is associated with post-discharge VTE and bleeding rates.

## Methods

### Data Sources

This retrospective cohort study used administrative claims data. Our data source was a 10 % random sample of the IQVIA LifeLink PharMetrics Plus database from July 2009 to March 2022. The IQVIA LifeLink PharMetrics Plus database is an administrative claims database representative of the commercially insured population of the United States with respect to age, gender, geographic location, and type of insurance coverage.^19^ The database includes in- and outpatient claims as well as retail and mail order prescription records and has been used extensively for pharmacoepidemiology research.^20,21^ The dataset includes an enrollment file that provides information regarding patient demographics, medical and pharmacy benefits eligibility, and insurance coverage. The claims files include in- and outpatient claims related to in- and outpatient care including diagnosis codes (ICD-9-CM before 1 October 2015 and ICD-10-CM after that), procedural codes (including CPT/HCPCS codes), provider specialty, cost, and discharge destination. Prescriptions claims also are included in the claims file and describe retail prescriptions filled including drug name, dosage, formulation, strength, route of administration, and generic product identifier, co-pay, and provider specialty.

### Study Design and Population

The study selected cancer patients who underwent one of the following complex GI operations based on Current Procedural Terminology (CPT) codes. Pancreas surgery included pancreatoduodenectomy (CPT 48150, 48152, 48153, 48154, and 48155), total pancreatectomy (CPT 48160), and distal/central pancreatectomy (CPT 48140, 48145, and 48146). Liver surgery included partial hepatectomy (CPT 47120), hemihepatectomy (CPT 47125 and 47130), and extended hemihepatectomy (CPT 47122). Gastric surgery included subtotal (CPT 43631, 43632, 43633, and 43634) and total gastrectomy (CPT 43620, 43621, and 43622). Esophageal surgery included transhiatal (CPT 43107 and 43286), Ivor-Lewis (CPT 43117, 43121, 43122, and 43287), and three-field (CPT 43112 and 43288) esophagectomy with or without interposition (CPT 43108, 43113, 43116, 43118, 43123).

A diagnosis of cancer was established based on claims using International Classification of Diseases (ICD)-9-CM and ICD-10-CM codes (Table 6) in the period from 6 months before the surgery to 1 month after the surgery. The study was reviewed and approved by the institutional review board at the University of Arkansas for Medical Sciences.

**Fig. 1:**
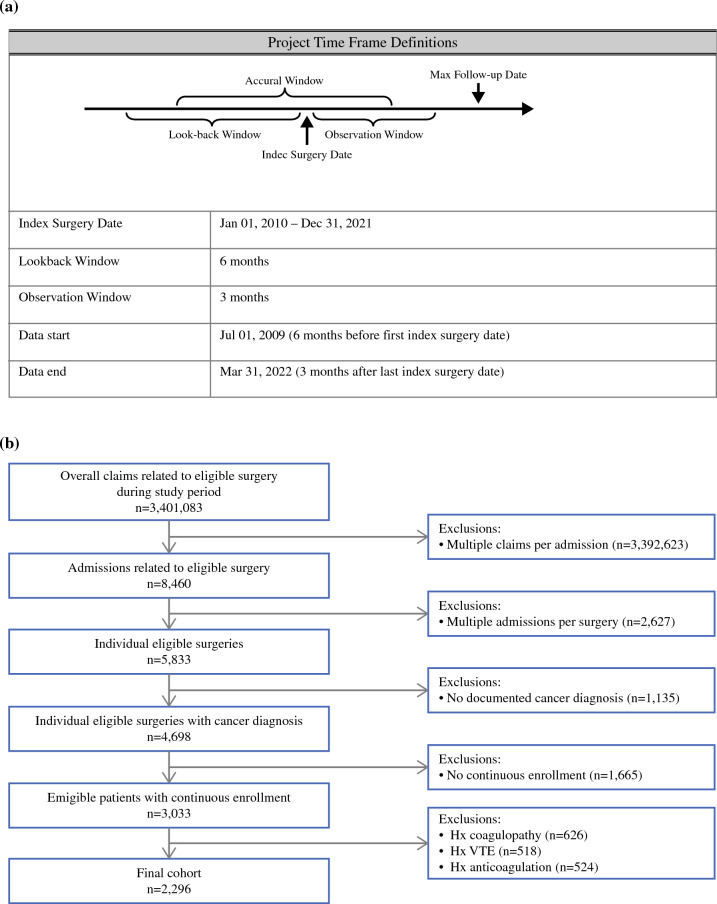
**A** Project time frame definitions. **B** Flow chart of the patient selection process.

The surgery eligibility window (index surgery date) was from January 2010 to December 2021. The look-back window, assessing comorbidities and applying inclusion and exclusion criteria, was 6 months before the index surgery through the date of surgery. The outcome assessment window started on the date of surgery and went through 90 days postoperatively (Fig. 1A). The study excluded patients who were not continuously enrolled for medical and prescription benefits throughout the entire study time frame (from 6 months before the date of surgery to 3 months afterward).

The exclusion criteria during the development of our cohort ruled out preexisting coagulopathy or history of VTE or bleeding (based on ICD-9-CM/ICD-10-CM codes) as well as prior receipt of anticoagulation (based on prescription claims). Patients who experienced postoperative VTE or bleeding while they were inpatients and those hospitalized 28 days or longer postoperatively during the index hospital admission (when the index surgery took place) were excluded from the final analyses because they were not eligible for extended VTE prophylaxis.

### Exposure

Exposure was defined as a prescription filled for prophylactic low-dose anticoagulation within 7 days after discharge from the index hospitalization during which the index surgery occurred. The agents and dosing of eligible anticoagulants are presented in Table 7.

### Outcomes

The primary outcomes of interest were the occurrence of post-discharge VTE and bleeding events during the 90 days after the index surgery. The study defined VTE as deep venous thrombosis (DVT) or pulmonary embolism (PE) with a primary diagnosis recorded in the inpatient, emergency department (ED), or outpatient setting, or resulting in prescription of therapeutic anticoagulation or insertion of an inferior vena cava (IVC) filter. Bleeding was defined as gastrointestinal (GI) bleeding or intracranial hemorrhage diagnosed in the inpatient or ED setting. Thromboembolic and bleeding events were based on the ICD-9-CM and ICD-10-CM codes (Table 6), which had been previously validated.^22^ Administrative claims data have been deemed an accurate source for identification of “clinically significant” events because these would invariably lead to a prescription, intervention, hospitalization, or death.^23,24^

### Covariates

Covariates were selected based on clinical relevance and included patient demographics (age as a continuous variable, sex), insurance coverage, surgery type and index year, prior prescriptions of anticoagulants, and overall measure of comorbidity burden as assessed by the Elixhauser index.^25^

### Statistical Analysis

Baseline characteristics and surgical outcomes are described using summary statistics. Categorical data are presented as totals (*n*) and percentages (%), whereas continuous data are presented as medians and interquartile ranges (IQRs). Discrete variables were compared using the Pearson chi-square test. Continuous variables were assessed using Student’s *t* test or the Mann-Whitney *U* test, as appropriate. Statistical significance was set at a *p* value lower than 0.05.

The patients who filled a prescription for extended VTE prophylaxis were compared with those who did not using multivariable logistic regression models. The first model identified characteristics independently associated with filling of an extended VTE prophylaxis prescription and included demographics, self-insurance coverage, type of surgery, and comorbidity. The second model estimated the effect of VTE prophylaxis on postoperative 90-day VTE and bleeding with adjustment for all covariates. Results were reported as adjusted odds ratios (ORs) with 95 % confidence intervals (CIs). The performance of each model was assessed using the area under the receiver operating curve (AUC). An ad hoc analysis was performed to explore the incidence of all 90-day postoperative VTE episodes (including inpatient events).

## Results

In the IQVIA data, 5833 operations were identified, 4698 of which were performed for patients with a cancer diagnosis (Fig. 1B). After application of the exclusion criteria, 2296 eligible cancer operations were identified. After analysis of the inpatient data, 227 patients were excluded from the final analyses due to development of inpatient VTE/bleeding or hospitalization longer than 28 days pertaining to the index hospitalization, which would render them ineligible for extended VTE pharmacoprophylaxis (Table 1).

**Table 1:** Operations excluded from the final analyses due to inpatient events (from 2296 to 2069 eligible operations)

Exclusion criterion	Pancreas surgery (*n* = 904) *n* (%)	Liver surgery (*n* = 694) *n* (%)	Gastric surgery (*n* = 361) *n* (%)	Esophageal surgery (*n* = 337) *n* (%)
Inpatient VTE	19 (2.1)	14 (2.0)	5 (1.4)	14 (4.2)
Inpatient bleeding	24 (2.7)	3 (0.4)	39 (10.8)	8 (2.4)
Hospital stay ≥28 days	49 (5.5)	13 (1.9)	30 (8.4)	48 (14.2)

*VTE* venous thromboembolism

### Population Characteristics

**Fig. 2:**
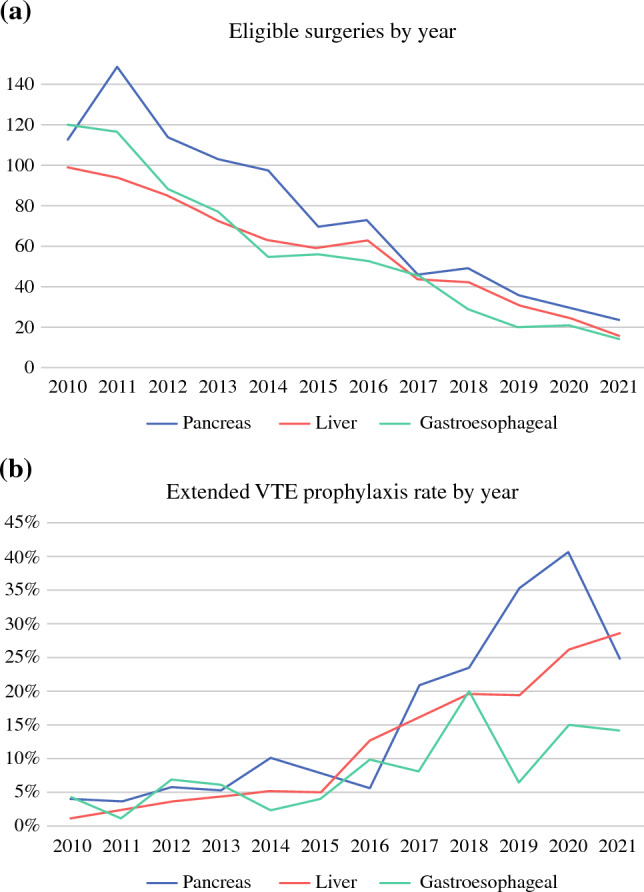
**A** Number of eligible individual operations by year. **B** Rate of individuals filling prescriptions for extended VTE prophylaxis after an eligible surgery, by year. VTE, extended venous thromboembolism

Due to decreasing numbers of recipients contributing to the data, the number of eligible surgeries in the provided data sample decreased over time during the study period, starting with 332 operations per year in 2010 and declining to 55 operations in 2021 (Fig. 2A). Of the remaining 2069 eligible patients, 176 (8.5 %) filled a prescription for extended VTE prophylaxis. This rate varied by type of operation (highest for pancreas surgery: 10.4 % during the study period) and increased over time (from 3.1 % in 2010 to 23.1 % in 2021; Fig. 2B).

The vast majority of the 176 patients who filled an extended VTE prophylaxis prescription received enoxaparin (*n* = 169, 96 %), and a few patients received apixaban (*n* = 3), dalteparin (*n* = 3), or rivaroxaban (*n* = 1).

The median age of the eligible population was 49 years, and 911 (44 %) of the patients were females. The study documented hypertension in 60.4 % and diabetes in 26.6 % of the patients, and the median Elixhauser index was 6 (IQR, 4–7). The median postoperative hospital stay during the index hospitalization was 7 days (IQR, 5–10 days) in both groups. The group receiving extended VTE prophylaxis was older (median age, 52.2 vs 48.7 years; *p* < 0.001), but the two groups were otherwise comparable in terms of comorbidities and insurance coverage (Table 2) except for the rate of prophylaxis among the self-insured patients (1.6 % for self-insured vs 9.6 % for other; *p* = 0.005).

In the multivariable analysis, only older age was independently associated with extended VTE prophylaxis (OR, 1.24; 95 % CI, 1.18–1.31). All U.S. geographic regions were equally represented in the dataset (East 24 %, West 26 %, Midwest 30 %, South 20 %), and region was not associated with extended VTE prophylaxis (*p* = 0.58).

**Table 2:** Baseline population characteristics

Characteristic	Extended VTE prophylaxis (*n* = 176) *n* (%)	No extended VTE prophylaxis (*n* = 1893) *n* (%)	*p* value
Median age: years (IQR)	52.2 (49.1–54.3)	48.7 (46.5–51.3)	<0.001
Female sex	83 (47.2)	828 (43.7)	0.66
Preoperative comorbidities			
Hypertension	108 (61.4)	1141 (60.3)	0.78
Diabetes	35 (19.9)	516 (27.3)	0.034
Kidney disease	10 (5.7)	153 (8.1)	0.26
Liver disease	84 (47.7)	850 (44.9)	0.47
Obesity	67 (38.1)	1421 (75.1)	<0.001
Elixhauser score	6 (5–7)	6 (4–7)	0.81
Insurance payer^a^			
Commercial	112 (67.5)	1,186 (65.0)	0.039
Medicare risk (advantage)	32 (19.3)	290 (15.9)	
Medicare cost (supplemental)	11 (6.6)	152 (8.3)	
Medicaid	9 (5.4)	72 (4.0)	
Self-insured	2 (1.2)	124 (6.8)	
Insurance product^b^			
Preferred provider organization	100 (61.4)	1,175 (65.5)	0.45
Health maintenance organization	46 (28.2)	458 (25.5)	
Consumer-directed health care	13 (8)	97 (5.4)	
Point of service	4 (2.5)	52 (2.9)	
Indemnity/traditional	0	11 (0.6)	
Type of surgery			
Pancreatectomy	87 (49.4)	746 (39.4)	0.038
Hepatectomy	54 (30.7)	610 (32.2)	
Gastrectomy	17 (9.7)	278 (14.7)	
Esophagectomy	18 (10.2)	259 (13.7)	

*VTE* venous thromboembolism; *IQR* interquartile range

^a^Insurance payer information was available for 1990 operations.

^b^Insurance product information was available for 1956 operations.

### Clinical Outcomes

After discharge, 108 of the patients (5.2 %) experienced VTE (48 DVT, 73 PE), and another 108 of the patients (5.2 %) experienced bleeding (64 GI bleeds, 25 all-cause transfusions, 3 brain bleeds, 25 other bleeds) within 90 days postoperatively. The findings showed no association between type of surgery and the rate of VTE (pancreas 6.2 %, liver 4.1 %, gastric 5.8 %, esophageal 4.3 %) or bleeding (pancreas 6.4 %, liver 3.8 %, gastric 6.4 %, esophageal 4.0 %). The rate of post-discharge VTE or bleeding did not differ between the patients who received extended VTE prophylaxis and those who did not (Table 3).

**Table 3:** Univariate analysis of post-discharge 90-day clinical outcomes

Outcome	Extended VTE prophylaxis (*n* = 176) *n* (%)	No extended VTE prophylaxis (*n* = 1893) *n* (%)	*p* value
VTE	12 (6.8)	96 (5.1)	0.32
DVT	4 (2.3)	44 (2.3)	0.97
Pulmonary embolism	9 (5.1)	64 (3.4)	0.23
Bleeding	7 (4.0)	101 (5.3)	0.44
Gastrointestinal bleed	4 (2.3)	60 (3.2)	0.51
Brain bleed	0	3 (0.2)	0.60
Transfusion	1 (0.6)	24 (1.3)	0.42
Other bleed	2 (1.1)	24 (1.3)	0.88

*VTE* venous thromboembolism; *DVT* deep vein thrombosis

In the multivariable analysis, extended VTE prophylaxis was not independently associated with either major bleed or VTE. Only the Elixhauser index was independently associated with the occurrence of 90-day VTE (OR, 1.24; 95 % CI, 1.15–1.34, Table 4) and bleeding (OR, 1.25; 95 % CI, 1.16–1.35; Table 5).

**Table 4:** Risk-adjusted association of extended VTE prophylaxis with post-discharge venous thromboembolism^a^

	Adjusted OR	95 % CI	*p* Value
Age	0.97	0.91–1.04	0.39
Female sex	1.21	0.81–1.80	0.36
Surgery type (reference: pancreas)			
Liver	0.70	0.43–1.14	0.16
Gastric	0.90	0.51–1.60	0.72
Esophageal	0.70	0.36–1.35	0.29
Elixhauser score	1.24	1.15–1.34	<0.001
Extended VTE prophylaxis	1.54	0.81–2.96	0.19

*VTE* venous thromboembolism; *OR* odds ratio; *CI* confidence interval

^a^A main-effect multivariable logistic regression model was developed for prediction of 90-day post-discharge VTE. The receiver area under the curve for the model was 0.66.

**Table 5:** Risk-adjusted association of extended VTE prophylaxis with post-discharge bleeding^a^

	Adjusted OR	95 % CI	*p* Value
Age	1.04	0.98–1.11	0.23
Female sex	0.67	0.44–1.03	0.066
Surgery type (reference: pancreas)			
Liver	0.66	0.40–1.08	0.097
Gastric	0.90	0.51–1.57	0.71
Esophageal	0.54	0.27–1.06	0.073
Elixhauser score	1.25	1.16–1.35	<0.001
Extended VTE prophylaxis	0.72	0.32–1.61	0.42

*VTE* venous thromboembolism; *OR* odds ratio; *CI* confidence interval

^a^A main-effect multivariable logistic regression model was developed for prediction of 90-day post-discharge bleeding. The receiver area under the curve for the model was 0.70.

**Fig. 3:**
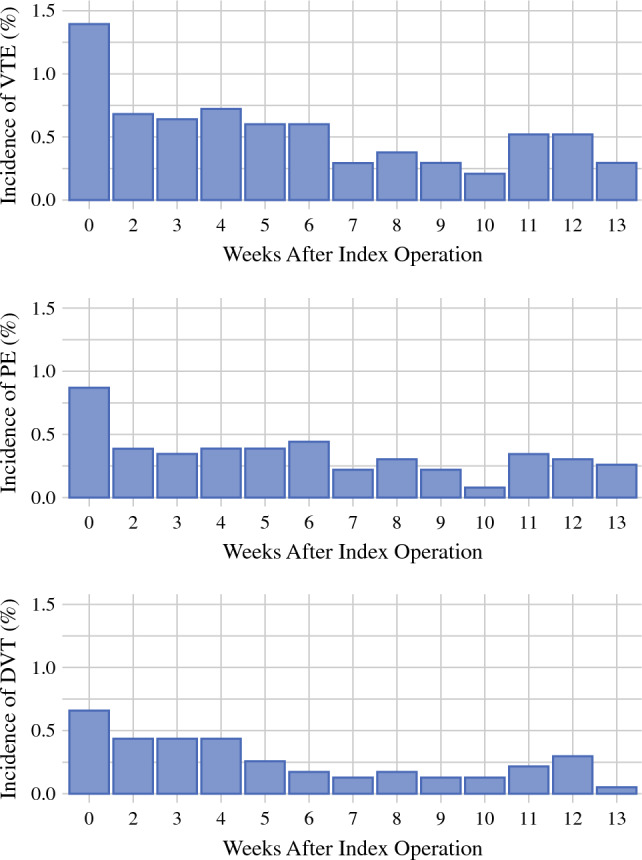
Rates of postoperative (including inpatient) venous thromboembolism occurrences by postoperative week.

In the ad hoc analysis of the 2296 initially eligible patients, the rate of VTE was highest during the first week postoperatively (1.4 %) and remained considerable (0.3–0.7 % per week) throughout the follow-up period (3 months). The postoperative rates of VTE, PE, and DVT (including inpatient events during the index admission) are presented in Fig. 3.

## Discussion

In this retrospective analysis of 2296 complex GI operations for cancer patients, the adherence to extended VTE pharmacoprophylaxis prescription guidelines was very low. Although the adherence increased over time, the majority of the patients undergoing surgery even in 2020–2021 did not fill an eligible prescription. The findings showed no difference in the 90-day post-discharge VTE or bleeding rates between the patients who filled an eligible prescription and those who did not. However, the statistical power of our analyses was limited by the relatively low number of events.

The current practice guidelines recommend extended VTE prophylaxis (4 weeks postoperatively) with LMWH for patients undergoing abdominal or pelvic surgery for cancer (recommendation grade 1B).^5^ This is based largely on three RCTs that used invasive contrast venography to screen for postoperative VTEs and were at least partially sponsored by the drug manufacturer. The Enoxacan II trial (multicenter, double-blind, placebo-controlled) randomized 332 patients to 4 weeks versus inpatient (6–10 days) postoperative subcutaneous enoxaparin (40 mg daily) and reported significantly higher VTE rates in the placebo group (12 % vs 4.8 % at 28 days postoperatively), mostly consisting of patients with distal DVT (10.2 % vs 4.2 %).^10^ The Danish trial (multicenter, assessor-blind, open-label) randomized 427 patients to 4 weeks versus 1 week of postoperative subcutaneous dalteparin (5000 IU daily) and reported significantly higher VTE rates in the short-course group (16.3 % vs 7.3 % at 28 days postoperatively), mostly due to a difference in the proximal DVT rate (8 % vs 1.8 %), but only 3 of the 343 evaluable patients were symptomatic.^11^ The CANBESURE trial (multicenter, double-blind, placebo-controlled) randomized 625 patients to 4 weeks versus 8 days of postoperative subcutaneous bemiparin (3500 IU daily) and reported no difference in the composite VTE outcome between the groups (13.3 % vs 10.1 % at 28 days postoperatively).^12^

In contrast, the current study relied on administrative claims for the diagnosis of VTE. As such, the events captured in our dataset were assumed to be clinically significant (however, rare instances of incidental findings on imaging in asymptomatic patients cannot be excluded). We noted an inpatient VTE rate of 2.2 % and a post-discharge 90-day VTE rate of 5.2 %, consistent with the literature.^26^ Interestingly, we noted a substantial number of new VTE events throughout the 3-month follow-up period. Although this has not been specifically investigated previously, preliminary evidence from readmission data^27^ and the Ontario Cancer registry^28^ suggest that the risk of VTE for cancer patients remains high for several months postoperatively.

This study has important clinical implications. We analyzed a large administrative dataset representative of the commercially insured U.S. population and analyzed more than 2000 pancreatectomies, hepatectomies, gastrectomies, and esophagectomies in cancer patients. In contrast to the existing RCTs with stringent inclusion/exclusion criteria and universal VTE screening of asymptomatic patients, this study represented real-world evidence and focused on clinically significant events. The large sample allowed for adequate statistical power and additional exploratory analyses.^29^

Our findings, interpreted in the context of the existing clinical trials and recent retrospective studies, suggest that extended VTE prophylaxis may not be beneficial for all cancer patients undergoing complex GI surgery. Our findings should be interpreted with consideration of certain limitations. First, the study was a retrospective analysis of a claim-based database, with the limitations inherent in such analyses (e.g., quality and granularity of data, potential sources of bias, type 1 error).^30^ Although the population in this database is representative of the commercially insured U.S. population, it may under-represent patients older than 65 years, diseases covered by government programs (e.g., end-stage renal disease), or diseases with specifically limited plan coverage (e.g., mental health services). In addition, data on patient race were not available, discharge destination was not examined, and the comorbidities as captured using the ICD-9-CM and ICD-10-CM classification may not have been optimal for risk adjustment.^31^ It also is possible that certain patients undergoing complex GI surgery had vascular reconstruction, which could have affected both their risk for VTE and the prescription of anticoagulation, and that certain patients were prescribed anticoagulation but did not fill the prescription (only filled prescriptions are captured in the data). There was potential for immortal time bias due to inclusion of only patients with 3 months of continuous enrollment postoperatively, although the effect of this likely was negligible. Despite control for demographics, plan characteristics and clinical characteristics such as comorbidity burden, residual confounding, and a relatively small sample may explain the null findings between VTE prophylaxis and bleeding and VTE events.

In conclusion, only a minority of cancer patients undergoing complex GI surgery are getting extended VTE prophylaxis postoperatively. In this study, receipt of extended VTE prophylaxis was not associated with post-discharge VTE or bleeding rates for commercially insured patients. Future research may define the risk for late post-discharge VTE and identify groups that will benefit from extended prophylaxis.

## Appendix

See Tables 6 and 7.

**Table 6:** ICD-9-CM and ICD-10-CM codes for the examined clinical outcomes and covariates

Diagnosis	ICD-9-CM	ICD-10-CM
Acute deep venous thrombosis	451.1, 451.81, 451.83, 453.4, 453.82, 453.83, 453.84, 453.85, 453.86, 453.87, 453.89	I80.1, I80.2, I82.2, I82.4, I82.60, I82.62, I82.A1, I82.B1, I82.C1, I82.90
Acute pulmonary embolism	415	I26
Gastrointestinal bleeding	455.2, 455.5, 455.8, 456.0, 456.20, 459.0, 530.21, 530.7, 530.82, 531.0, 531.2, 531.4, 531.6, 532.0, 532.2, 532.4, 532.6, 533.0, 533.2, 533.4, 533.6, 534.0, 534.2, 534.4, 534.6, 535.01, 535.11, 535.21, 535.31, 535.41, 535.51, 535.61, 535.71, 537.83, 537.84, 562.02, 562.03, 562.12, 562.13, 568.81, 569.3, 569.85, 569.86, 578	I85.01, I85.11, K20.81, K20.91, K21.01, K22.11, K22.6, K25.0, K25.2, K25.3, K25.4, K25.6, K26.0, K26.2, K26.3, K26.4, K26.6, K27.0, K27.2, K27.3, K27.4, K27.6, K28.0, K28.2, K28.3, K28.4, K28.6, K29.01, K29.21, K29.31, K29.41, K29.51, K29.61, K29.71, K29.81, K29.91, K31.811, K31.82, K55.21, K57.01, K57.11, K57.13, K57.21, K57.31, K57.33, K57.41, K57.51, K57.53, K57.81, K57.91, K57.93, K62.5, K66.1
Brain bleeding	430, 431, 432, 852.0, 852.2, 852.4, 853.0	I60, I61, I62, S06.34, S06.35, S06.36, S06.37, S06.38, S06.4, S06.5, S06.6
Other bleeding	423.0, 459.0, 593.81, 719.1, 784.7, 784.8, 786.3, 599.71	I31.2, M25.0, R04, R31.0, R58
Elixhauser Index comorbidities (including cancer)^a^	University of Manitoba SAS code	University of Manitoba SAS code

*ICD* International Classification of Diseases; *CM*, clinical modification

^a^University of Manitoba SAS macro code for Elixhauser Index:

http://mchp-appserv.cpe.umanitoba.ca/Upload/SAS/_ElixhauserICD9CM.sas.txt (last accessed February 20, 2023); http://mchp-appserv.cpe.umanitoba.ca/Uload/SAS/_ElixhauserICD10.sas.txt (last accessed February 20, 2023)

**Table 7:** List of eligible anticoagulants^a^

Drug name	Prophylactic dosage (mg)	Therapeutic dosage (mg)
Enoxaparin	30, 40	60, 80, 100, 120, 150, 300
Dalteparin	2500 U, 5000 U	7500 U, 10,000 U, 12,500 U, 15,000 U, 18,000 U, 25,000 U, 95,000 U
Tinzaparin	N/A	Any
Fondaparinux	2.5, 5	7.5, 10
Warfarin	N/A	Any
Apixaban	2.5	5
Rivaroxaban	2.5, 10	15, 20
Dabigatran	110	75, 150

*mg* milligram; *u* units; *N*/*A* not applicable

^a^Prophylactic dosage was used for assessment of outcomes and therapeutic dosage for application of exclusion criteria (history of prior anticoagulation). Frequency of administration was not evaluated due to limitations on the data.

〹
